# Artificial intelligence in psychiatric care and education: a qualitative study of factors influencing adoption in Singapore

**DOI:** 10.5116/ijme.6a7f.60c5

**Published:** 2026-08-26

**Authors:** Daniel Poremski, Ker-Chia Wei, Boon Tat Ng, Jia Wang, Kang Sim

**Affiliations:** 1 Institute of Mental Health (IMH), Singapore, Republic of Singapore

**Keywords:** Continued education, psychiatry, thematic analysis, artificial intelligence integration, Singapore

## Abstract

**Objectives:**

This study aimed to understand how AI's perceived role interacts with emerging barriers and facilitators to identify factors influencing its adoption across clinical and educational professions in Singapore.

**Methods:**

This study followed a qualitative approach guided by a medical-pedagogical theoretical framework. Semi-structured interviews were conducted between May and July 2025 and followed an interview guide based on the medical-pedagogical framework. Twenty-four people participated, including eight nurses, six psychiatrists, and 10 allied health professionals. All were clinicians and educators. Data were analysed using thematic analysis, with attention to emergent patterns across patient care and educational contexts.

**Results:**

Participants recognised significant potential for AI in patient care and healthcare professions education, particularly for information access, retrieval, clinical documentation, AI-augmented training methods such as virtual patients and educational content creation. Barriers included fears of professional skill degradation, role confusion, lack of familiarity with capabilities and the need for personal evidence of benefit. Enablers encompassed integrated, context-specific training, clear governance frameworks, and peer networks facilitating experiential learning and responsible use. Participants emphasised maintaining human connection and reflective practice as essential to psychiatric care and education.

**Conclusions:**

Effective AI adoption in psychiatric care and education can be facilitated by clearly delineating tasks, embedding digital literacy into training, communicating robust governance structures, and introducing peer-led communities of practice. Relevant stakeholders should be engaged to align AI deployment with real clinical workflows, optimising both patient care and educational outcomes.

## Introduction

Artificial intelligence (AI) is expanding at a rate that surpasses human comprehension and that dwarfs what David M Eddy observed about modern medicine’s complexity decades ago.^1^ It would appear that every week news emerges of a new industry benchmark,^2^ with perpetual jockeying for model superiority.^3^ The medical sciences have not been spared from this deluge and several technologies are emerging that propose to facilitate medical practice and education.^4^^-^^8^ Precision medicine, supported by pharmacogenomics and AI, promises to automate and streamline clinical workflows, enabling clinicians to make optimised decisions. However, clinicians must retain and enhance core competencies rather than becoming entirely dependent on technological aids.^4^^,^^6^^,^^9^ The transparency of these tools is vital to their adoption, governance, and utility.^10^^-^^12^

Psychiatric settings present distinctive challenges for AI integration that differentiate them from other medical specialties.^13^ The therapeutic relationship and human connection may be more fundamental in psychiatry than in any other medical field.^14^^-^^17^ Quantitative indicators and standardised metrics need to be thoughtfully interpreted,^18^ even whilst language models may appear to offer comprehensive solutions to complex psychological phenomena.^3^ Additionally, psychiatric care inherently operates through interprofessional collaboration, involving psychiatrists, psychologists, nurses, allied health professionals such as pharmacists, medical social workers, psychologists, case managers, occupational therapists who jointly contribute to both direct patient care and health professions education (HPE). Uniform approaches to AI applications risk overlooking setting-specific requirements, leaving professionals vulnerable to applying generalised guidelines to specialised contexts.^19^ As technological advancement accelerates, educational stakeholders require discipline and context specific guidance to determine relevant content for training and practice.^8^^, ^^20^

Despite the accelerating integration of AI technologies into healthcare and education, there exists a knowledge gap regarding interprofessional perspectives within psychiatric care settings.^13^ Whilst significant investment continues in technological development, empirical research examining the views and concerns of diverse interprofessional groups about AI's role in patient care and HPE remains limited. This paucity of evidence-based understanding of ground-level perceptions may explain why the translation of experimental evidence into clinical practice continues to falter, despite promising early results.^13^^,^^21^  The absence of evidence constrains the development of targeted strategies and support mechanisms that could facilitate effective, context specific AI adoption within psychiatric context.^22^ Without comprehensive insight into how various interprofessional team members perceive AI's clinical and educational applications, institutions risk implementing generic approaches that may not address professional or contextual needs.

This study seeks to obtain interprofessional perspectives on the integration of AI into psychiatric care and education to (1) understand its perceived role in patient care and HPE; and (2) to understand facilitators and barriers to its adoption. Understanding these perceptions and identifying needs will provide valuable insights for developing targeted resources and strategies.

## Methods

### Study Design and Setting

This study followed a qualitative approach guided by a medical–pedagogical framework to describe themes related to the adoption of AI technologies and their integration into psychiatric care and health professions education. Its participants were selected from Singapore’s national centre for tertiary psychiatric care and training, the Institute of Mental Health. This institute hosts the pre-professional training of several healthcare occupations, as well as postgraduate training for psychiatric residents and fellowships. The pre-professional education involves clinical rotations of over 3000 learners annually across medical, nursing and allied health professional groups. The National Psychiatry Residency Program, hosted at the institute, is accredited by the Joint Commission for Specialty Training under the Ministry of Health, Singapore. A large proportion of its teaching faculty holds joint teaching appointments with local universities.

### Theoretical framework

The widespread application of AI has generated numerous potential frameworks for structuring data collection and analysis across diverse domains including ethics, computer science, law, medical policy, and philosophy.^23^ However, frameworks specifically addressing patient care and pedagogy within psychiatric contexts remain limited. Given this study's dual focus on understanding AI's role in healthcare and HPE, we selected a medical perspective as the foundational framework whilst incorporating pedagogical principles as necessary. Our approach employed a predominantly deductive methodology that integrated complementary pedagogical theory with allowances to capture emergent themes across clinical and educational domains.

The theoretical foundation drew upon an integrative literature review of AI implementation guidelines in medicine,^11^ Singapore's national AI governance framework issued by the Ministry of Health (AIHGle), and mental-health-specific reviews addressing implementation challenges.^5^ These sources highlighted challenges related to transparency, explainability, and reproducibility. Crossnohere and colleagues^11^ emphasised ethics, effectiveness, and engagement, whilst Singapore's AI governance framework prioritised fairness, responsibility, and patient-centredness. This scaffolding provided a structured lens for examining interprofessional perspectives on AI in patient care and HPE, as well as perceived barriers and enablers to AI adoption.

We used the Consolidated criteria for reporting qualitative research to structure the manuscript^24^ with reference to Wu, Wyant.^25^

### Participants and Sampling

To address our research aims, we targeted three distinct professional groups: physicians, nurses, and allied health professionals (including pharmacists, psychologists, case managers, occupational therapists and medical social workers) within a national tertiary psychiatry care setting. Whilst existing literature examines these groups independently, we elected to organise participants according to their dual responsibilities in clinical care and educational practice rather than strict professional boundaries, recognising the integrated nature of patient care and interprofessional development.

We identified key informants fitting these criteria: (1) active involvement in psychiatric service provision; (2) current role in HPE (including clinical supervision, teaching, mentoring, or curriculum development); and (3) demonstrated interest in AI as it relates to both clinical care and educational practice. We excluded individuals whose AI interest was limited to personal applications.

Recognising potential heterogeneity within professional groups as a source of diverse perspectives, we recruited up to eight participants per group, targeting 24 participants in total. Interview and recruitment proceeded concurrently, as themes emerging from one professional group were not expected to influence the line of questioning of another, particularly given the dual focus on patient care and educational applications. We aimed for thematic saturation, the point at which no new content about the themes of interest emerged following additional interviews, within each professional group across both domains of inquiry. Thematic saturation occurred after six interviews for the nursing group, eight for the psychiatrist group, and 10 for the allied health group.  Recruitment occurred from May 2025 to July 2025. No person approached for participation in the project refused the invitation.

The study received ethics approval from institutional and national ethics review committees (Institutional Research Review Committee 893-2025, National Health Group Domain Specific Review Board Domain A 2024-4540). Participants gave written informed consent. A remuneration of $50 was provided to participants. The study complied with the Helsinki Declaration and all local personal data protection policies.

### Data collection

Our semi-structured interviews followed an interview guide developed from our theoretical framework, specifically designed to address both research aims across patient care and HPE contexts. The interview contained: a biographical section establishing participants' professional backgrounds and roles; a section on AI-familiarity covering clinical and educational applications; exploration of participants' perspectives on AI's role in care within psychiatric settings; examination of AI's perceived role in HPE; and identification of enablers and barriers to AI adoption in healthcare and HPE.  Interview prompts were strategically designed to facilitate the analysis of content primacy, providing opportunities for participants to articulate their priorities before introducing specific examples. The first author conducted all interviews.

Automated transcription utilised a locally developed government-based transcription tool, with ten-point accuracy checks conducted through random sampling of interview segments. Any sections containing transcription deviations were manually transcribed verbatim by study team members. Common transcription errors involved incorrect speaker attribution, punctuation mistakes, or misrecognition of "ChatGPT." Interviews averaged 64 minutes with a standard deviation (SD) of 8 minutes. One participant declined audio recording.

The interviewer documented post-interview memos highlighting themes and assessing rapport. Of 24 interviews conducted, the interviewer had pre-existing relationships with nine participants, whilst three participants took time to warm up to the interview method. Although some prompts evolved throughout the interview process to better capture nuances, the core interview guide remained unchanged.

### Analysis

Thematic analysis was selected as the most appropriate analytical approach to achieve our dual study aims.^26^^, ^^27^ The first author, who conducted all interviews, performed initial coding whilst building upon post-interview memos, systematically examining themes across both clinical care and HPE contexts. Codes were generated first deductively, following the project’s theoretical framework. For example, the framework highlights that transparency, explainability, and reproducibility likely exist as important elements of consideration but may also hinder the implementation of AI tools when they are poorly addressed during deployment of a technology. Thus, these three were coded. However, the team was sensitive to content that fell outside of the deductive coding list, and as new topics emerged inductively, they were progressively added to the coding list. At a final coding stage, all interviews were recoded with the updated coding list. Regular team meetings facilitated theme discussion and resolved coding uncertainties, ensuring thematic consistency and shared understanding across the research team. These meetings allowed the team to group the codes into themes. The team then discussed the relationships between the themes to generate a parsimonious explanation for their interdependence. The team also ensured that the analysis proceeded in a way that would allow for the generation of relevant recommendations.

We considered both primacy and frequency of codes across patient care and educational contexts. For instance, 21 participants initially expressed concerns about language model accuracy in clinical applications, warranting priority attention, whilst educational-specific concerns and less frequent topics mentioned mid-conversation received proportionate consideration within their respective domains. Special attention was given to novel themes not previously reported in literature across either patient care or educational contexts, applying a "novelty consideration" to augment traditional primacy, intensity, and frequency criteria.

To enhance transparency and trustworthiness, themes are supported by extensive quotations with participant codes enabling traceability across both clinical and educational findings.^28^ We verified all quotations against original recordings and edited them to remove filler words whilst preserving meaning. Analysis was completed using NVivo 11, without the aid of AI.

### Reflexivity

No team members possess computer science expertise or financial interests in AI tool development or licensing. While this study did not involve patients or members of the public in its study design team or in the discussion of its conception and execution, the study team contains representation of relevant stakeholder groups, including physician educators, nurse educators and allied health educators, providing suitable input to account for considerations about recruitment, design, and result dissemination. The first author served as sole interviewer, bringing extensive experience in mental health service research in Singapore and contributing to local AI policy development across both clinical and educational contexts. His generative AI knowledge is academic rather than technical, positioning him as an early-to-middle adopter^29^ who regularly engages with AI developments through academic channels whilst remaining mindful of potential technology misrepresentation in both clinical and educational applications. As an early-to-middle adopter, he was mindful not to lead participants into territory they had not voluntarily shared. This meant calling on his experience to build rapport with those who were enthusiastic and avoiding dismissive responses to participants who downplayed AI advances or over-emphasized its limitations.

Given pre-existing relationships with nine staff members who work across clinical and educational roles, the interviewer maintained clear role boundaries, ensuring conversations remained focused on research objectives rather than institutional AI implementation discussions. This approach helped him avoid judging participants' AI usage legitimacy or feasibility.

During project conceptualisation, we established clear exclusion boundaries for administrative AI applications and personal usage, focusing specifically on patient care and HPE contexts along our research aims. Initially, we anticipated that targeting AI-interested participants might exclude perspectives of reluctant adopters across both domains. However, this assumption proved incorrect when many currently interested participants revealed experiencing initial AI resistance during their literacy development. This enabled exploration of both voluntary and professionally mandated adoption perspectives relevant to understanding barriers and enablers across clinical and educational applications.

## Results

We approached, recruited and interviewed 24 participants representing diverse professional groups within psychiatric care settings. A third of participants described themselves as early adopters of AI and technology in general, a third positioned themselves in the middle category (either early middle or late middle adopters), and the remaining considered themselves as lagging adopters. This distribution emerged naturally without intentional sampling. Only one participant characterised himself as a non-adopter. When asked about their definition of AI, 18 participants (75%) mentioned generative AI as their primary or sole conceptualisation. Two spoke first about machine learning, whilst the remaining demonstrated a balanced understanding encompassing large language models, robotics, machine vision, and learning algorithms.

Participants worked for the host institute an average of 13 years (SD 6 years). Eight (33.3%) were nurses, six (25%) were psychiatrists, and two each were case managers, medical social workers, occupational therapists, pharmacists, and psychologists. Only one participant had previous brief experience in an information technology field. Fourteen participants (58.3%) were men. Whilst we did not request precise ages, most participants were in their 40s, followed by those in their 30s.

The interplay between AI's role in patient care and HPE revealed interconnected considerations that transcended domain boundaries, with participants demonstrating relatively similar perspectives regardless of whether AI applications were oriented towards clinical practice or educational deployment especially related to barriers and enablers. Below we present the roles staff envisioned for AI across both activities, explain the common interconnected barriers and enablers, and conclude with practical implications to address these issues.

### Roles for AI in Patient Care and Health Professions Education

#### Patient Care Applications

In clinical contexts, participants utilised AI primarily to accelerate information retrieval and supply structured information and ideas for patient care decisions. Within multidisciplinary team settings, AI enabled staff to access information traditionally reserved for specific disciplines, potentially enhancing interprofessional collaboration and reducing patient waiting times.

“When I attend to the patient, the moment the patient asks for medication, "oh medication, you have to go to the pharmacist". "Oh, I got the nurse to attend to you". You know, then I got to get a nurse to come then, so there will be waiting time because the nurse is busy, you know, and then it's also human resource spending, because then you got to mobilise one more staff to explain. [...] Why? Because I have no knowledge and I have a lack of confidence to provide you with some of the basic information? So I think it's a wonderful idea if you are able to have AI to give us some of the foundational and functional knowledge of other discipline, so that when patient comes to us, or even caregivers, we are able to support them with some of the very common kind of needs that they are presented with.” (81011, early middle adopter, medical social worker)

Allied health staff conducting therapy sessions employed transcription and large language model tools such as Scribe to support post-session documentation and case formulation. These tools transcribed audio recordings. Everything is written down whilst providing session summaries and therapeutic feedback, enabling clinicians to focus more attention on patient interaction during sessions and reduced documentation burden.

“Yeah, so we use Scribe, right, so in Scribe you actually can tell Scribe: "please give me a four-piece formulation", and it gives you based on the transcripts that you had that day. The challenge, the limitation of Scribe is because every transcript is independent. It can't pull transcripts across sessions.” (81015, early middle adopter, psychologist)

Both applications were supported by government-developed tools capable of processing certain types of patient-level data. However, most participants noted using commercially available alternatives such as ChatGPT due to superior performance characteristics. Only one physician reported using a purpose-built advanced natural language processing platform reserved for medical practitioners through subscription, with a second discussing the need for institutional financial support for such resources.

No participant had used or prescribed AI to augment therapeutic interventions, though awareness of such technologies existed. Discussion of these technologies frequently included deployment concerns and requests for professional guidance:

“If they're bringing you information like: "hey, I did this because AI told me this, is the right thing to do?" It's quite straightforward, because doesn't matter where your source is. Could be your uncle [...] it could be AI. You just need to kind of weigh it against your clinical experience and opinion and then give a response that is medically valid. [...] I think what changes is a patient can kind of ask you: "should I talk to AI when I feel lonely? Should I try and get my AI to be my CBT therapist? Because I can't afford therapy". How do you respond to that? How do you say yes or no? What caution do you give them? Right, or do you just say: "I don't know".” (81021, early adopter, psychiatrist)

Similarly, whilst decision support tools were known to participants, they were not integrated into psychiatrists' clinical repertoire. These dilemmas underscored the uncertainty with which participants perceived the evidence supporting such tools, an important element elaborated upon in the barriers section below.

### Health Professions Education Applications

In educational contexts, participants perceived AI as a tool to innovate teaching approaches and enhance learning engagement and experience. Virtual patients, created internally with large language model assistance, were viewed as mechanisms for students to obtain exposure to clinical scenarios that could otherwise only be delivered through direct patient interaction.

“Using AI for clinical practice, so they will actually ask the AI questions, and then an AI-generated voice replies, so I think it helps them prepare for exams, so that could be something that we try to adopt in terms of how we train our residents [...] because I think it is something that's convenient, so with our traditional way of doing clinical practice, we need to hire a standardised patient to come down, and then everyone's calendars and schedules have to fit, but with the AI system, I think it's more convenient, so they could be practising it even at home, […] as long as they have a quiet space, they could be doing their practice, so I think it helps with convenience, logistics.” (81024, early middle adopter, psychiatrist)

These educational applications could bridge the gap between textbook case studies and simulated discussions for undergraduate learners, whilst also helping qualified clinicians familiarise themselves with specific scenarios such as forensic risk assessment. Educators additionally employed AI tools to generate examination questions, develop training materials and syllabi, and, similar to clinical contexts, as sources of content ideas for curriculum development.

### Barriers to AI Adoption Across Patient Care and Health Professions Education

Barriers could be organised sequentially, beginning with concerns about professional stability and role confusion, linked to unfamiliarity with technical capabilities, confusion about governance frameworks, and culminating in demands for evidence and personal proof-of-benefit across clinical and educational contexts.

### Professional Skill Degradation and Role Confusion

Depending on perspectives regarding AI task accomplishment, some participants perceived these tools as potentially replacing certain professional functions, leading 13 participants to express concerns about skill degradation in clinical acumen, educational knowledge, or supervision quality, if over-relied upon. This prompted most to consider which responsibilities should remain within their professional domain versus those that might be delegated to AI systems.

Allied health staff, who engaged more extensively in talk therapy than other professional groups, verbalised concerns about role security and professional identity more frequently than other staff types. One supervisor articulated these concerns from a management perspective to illustrate how such fears might affect the entire profession:

“It [AI] won't make them think, then they don't grow professionally. I'm not so worried about the part of taking the jobs, but it's like they don't need to think they don't need to grow professionally, because AI does everything for them. Then they become like, I don't know, almost like just robots. Doing for AI, what AI tells them to do, and I'm worried about the professional growth.” (81012, late adopter, medical social worker)

Alongside professional skill degradation concerns, some participants worried that AI transcription might remove reflective benefits inherent in manual notetaking, particularly in therapy-related work:

“So I should be jumping right at anything that would save me this time, but I think the way I approach my documentation and case note writing is I use the time to reflect a lot of my cases. I use that time to process what has happened in the session. You know what my clients say, what I responded with. I don't think that could be done for me fully by Scribe [AI].

Ya, so they would maybe get to a stage where they can make a really nice concise case note, but the part where I'm still reflecting on my practice and what I would do differently or what I will do next session. There's still very much human. I wonder if the AI can get to a stage next time where it would analyse your session and then say: "Okay, next session. This is the plan,[...] and these are my suggestions". That will be also creepy.” (81016, middle late adopter, psychologist)

Participants clearly believed that healthcare and education retained essential human elements that could not be reduced or replaced by AI tools:

“These are, these are human factors that no matter what AI system says in words or expresses to you, it just can't replace that human connection part. So I would say that healthcare system and healthcare education, it will always be that there will always be a component of working with tools, but you cannot replace the human component in both healthcare and education, in my own personal opinion.” (81011, early middle adopter, medical social worker)

Finally, the broader access to information provided by AI models could blur professional boundaries between interprofessional team members, contributing to role confusion if implementation did not consider the entire team:

“It's hard to think interdisciplinary, although I know there is a move towards that as well, but then I think to move that [way] we need to be quite firm and clear on our role first before, or at least our profession, before we start going inter-discipline. At least that's what I feel, because sometimes some boundaries can blur.” (81009, late adopter, occupational therapist)

### Lack of Familiarity and Desire for Co-creation

Whether threats to professional roles were genuine or perceived, they influenced staff intentions regarding AI adoption across both contexts. Participants felt that clear information about technical capabilities, limits, and institutional plans could reduce anxiety and support more deliberate use. Several noted confusion about who was leading AI efforts and how to access endorsed tools.

“So, actually there's a few major players right now. I can't quite identify that as a main driver, because everybody is moving their own interests and agenda forward at the same time. So I would say that gets a little bit confusing as well, because different people are doing different things and, there's no, or at least to my knowledge, there's no centralised communication effort to let us know[...] who is going to front and spearhead. Who do you contact? You know, so that gets a little bit confusing in some sense.” (81011, early middle adopter, medical social worker)

Participants also shared about the place of co-creation of training with staff to ensure relevance. They advocated for expert-led, context-specific sessions that address both clinical and educational use cases, with a view to cascading knowledge within departments.

“I want to let him [AI lecturer] know what we do and what I would like him to share with our people, rather than he come in and tell us [about] AI that's not relevant. So I think I need to let him understand what we do as a [hospital] social department, and maybe you know the curriculum development part. I need to co-work. Co-create with him rather than he come in and tell me what is AI.” (81012, late adopter, medical social worker)

### Wanting to See Evidence Personally

Whether concerning clinically deployed tools such as case summarisers or therapy adjuncts, or educational tools such as virtual patients or training aids, participants relied heavily on personal experience with tools to determine comfort levels for endorsing deployment across both contexts.

“I think if it really gets to a level... okay right now I've not encountered that before, but if I had moments more moments of saying "Wow, you know, actually, I would say that as a therapist" and if I notice that more and more ya, then I think we can look at that as an emerging pattern.” (81016, middle late adopter, psychologist)

This strategy had potential to negatively influence AI opinions when errors or hallucinations were encountered:

“The worst experience was just like when I desperately needed to run like some data analysis. Then it just wouldn't. It didn't even know how to answer that question. So that that was very frustrating, but I bet if I tried asking it now again, it might be very different from that time when I tried to use it, and it was very clear that it did not know its stuff. So, I remember, I remember being very angry, like even the AI cannot help me, [...] so that didn't make me like a super huge fan.” (81016, middle late adopter, psychologist)

### Enablers for AI Adoption Across Patient Care and Health Professions Education

#### Training and Integration into Learning Activities

Integrating AI use into training and critical thinking activities could also address the weight participants placed on firsthand AI tool experience. Some clinicians had integrated virtual patients into student supervision, jointly interacting with virtual patients to model basic interviewing and information gathering methods whilst highlighting tool limitations. This approach could prepare staff and students for hallucinations and invalid AI output, preventing negative experiences from unduly influencing practice and adoption.

“So, I was just being sort of like, trying to quickly quickly to get that.[...] You know, "please help me with an inspirational quote". But in the end it turns out that when I was checking it, actually, the inspirational quote and the person who quoted it. It was wrong, so so so so even something basic like this again, sort of like just reminds me that you know, look, we do really have to check it. And in in this case, for example, it was just a small thing, because if I've misquoted someone, you know, it's probably fine, but sort of like misquoting some information that could be, you know, having quite a serious impact in a clinical setting would be disastrous lah, so whichever we use, we would try to definitely check it.” (81028, middle late adopter, psychiatrist)

During student training, supervision, and continued staff education, some participants noted that active use of generative AI output could be beneficial as a useful object of critique, though they also worried about over-reliance.

“So for allowing students to use AI for assignment, it's still debatable which two views you see. [...] It's kind of a common things for the student to use AI to generate assignments so they can exercise their critical thinking by appraising whatever come out from the AI tools. So there is one perspective, but there the student will lose the ability to use AI. The ability to gather information, to synthesise the information together. So that could be something the students lose out in future. So, my own personal view is to be more cautious.” (81020, late adopter, nurse)

### Guidance and Governance

Guiding staff on appropriate AI use surfaced repeatedly across different contexts. Governance as an enabler emerged predominantly because it allowed the users to have confidence in the tool and signalled the institute’s support of its use:

“I think it still comes down to consensus, so I think if we can get like the department, for example, everybody to agree that we're going to try using this out and roll it out, then I think that would help in terms of like my confidence in using it, so it's not just my, my unilateral decision, but that like you know, the department or the hospital has decided that we're going to include this in part of our work workflow.” (81024, early middle adopter, psychiatrist)

Participants perceived guidance as fragmented or lacking across both healthcare and educational sectors. Without the guidance, they relied on their own judgement, which was not what they preferred:

“Participant: So they [patients] also told me: “Ya. I have scribe Too. Why can't you and I both turn on our scribe? At the same time you have your copy? I have my copy too”. Then to me is like, is that even allowed? You know, is that possible? Can a client turn on their own scribe and record the session as well, so we don't have rules around that, but I have told the client know you know you, you, you can't record our session. Yeah, so so I think that's where I find sometimes a bit tricky to navigate, and I'm just using my own impression, my own clinical judgement, and what I think is best for that client based on their presentation, which may not apply to every client. Also, then I may have to keep changing my approach depending on the presentation, but something a little bit more, I guess, organisational based might be helpful if there are certain guidelines.” (81018, early adopter, nurse)

Without the required governance, they expected that any managerial reaction to its malfunction would be deleteriously restrictive.

“If something does go amiss or haywire, I think it will set us back quite a fair bit because we are still quite new to AI and we have not established enough trust in its credibility.” (81011, early middle adopter, medical social worker)

### Peer Networks and Communities of Practice

Participants highlighted peer networks as powerful enablers of AI adoption. Colleagues who had experimented with tools were often seen as more trusted and persuasive than formal institutional communications. Informal sharing of prompts, use cases, and “dos and don’ts” across professions helped staff translate abstract AI policies into practical workflows in both clinical and educational settings.

“The peer would help you recognise the challenges related to clinical work, […], or related to the way that you have to integrate into your existing corpus of knowledge, more of like this, like the, I guess, like the nuances, so I think the tech expert might be able to help with the overview, but the peer might actually experience some of the things that you are, […] Like how to do certain things in clinic like that may not have been covered in detail, so having a peer who has already done it for a few times and is more experienced, come and you know, sit you through and say “this is what you do”, and take screenshots and show you.” (81024, early middle adopter, psychiatrist)

Participants described emerging communities of practice, formal and informal, in which clinicians and educators compared experiences, troubleshot problems, and co-developed norms for responsible use. These networks were viewed as critical to sustaining momentum beyond introductory workshops or top-down directives.

“I think it's really the time spent to help them understand and recognise that this helps, how you can show the efficiency, because once you do that well, all the more they'll use it again. It’s that one good experience to push them towards what you want them to do.” (81009, late adopter, occupational therapist)

## Discussion

This study examined interprofessional perspectives on AI within a psychiatric care environment, focusing on both clinical care and health profession educational domains. Participants envisaged AI roles in both healthcare (e.g., information retrieval, documentation, interdisciplinary support) and HPE (e.g., virtual patient simulations, curriculum generation). While many of these roles echo what appeared in our theoretical foundation^11^ and elsewhere,^22^^,^^30^ we have been able to link their occurrence and interplay with barriers. Key barriers included concerns about professional skill degradation, role confusion, limited familiarity and institutional policy opacity. The need for personal proof of benefit also emerged as an obstacle to its integration into clinical care and educational activities. Key enablers included targeted and contextualized training embedded in practice, clear governance and peer networks for knowledge exchange.

Beyond confirming interprofessional perspectives within psychiatry, this study contributes in three specific ways. First, it provides a dual-domain analysis demonstrating that perceptions of AI adoption are interdependent across psychiatric care and education, rather than operating in isolation. Second, it identifies an interrelated pattern of barriers and enablers, spanning professional, organisational, and experiential levels, rather than treating these as discrete factors. Third, it highlights context-specific insights in psychiatric and interprofessional settings, including concerns about erosion of reflective practice, blurring of professional boundaries through AI-enabled knowledge access, and the continued centrality of human connection in psychiatric healthcare.

In the clinical care domain, participants described AI as supporting rather than replacing human expertise. They reported uses in accelerating information retrieval, cross-discipline knowledge sharing within interprofessional teams, and transcription/summarisation to reduce documentation burden, especially in allied health therapy settings. While no participant reported using AI as a therapeutic agent or decision-making support, the emphasis on workflow support resonates with recent findings in mental health and medical AI literatures. For example, a scoping review of AI in mental health care identified that many tools currently support assessment, monitoring or education rather than fully automated therapy.^31^ The data suggest that, in psychiatric interprofessional settings, the value of AI lies in augmenting clinician capacity and streamlining tasks rather than supplanting core client-clinician relationships. It further highlights the importance of situating AI within existing workflows rather than disrupting them. Recognising these layered barriers is critical to designing implementation strategies that address more than just AI tool proficiency.^32^

Concerning education, participants saw AI as enriching learning experiences by facilitating the creation of virtual patients and other curriculum content and facilitating on-demand practice. These uses align with emerging educational research advocating for AI literacy, simulation, and scaffolded integration of AI tools into curricula of health professions education.^33^^,^^34^ However, participants also expressed caution: over-reliance on AI for document generation or simulation could reduce reflective practice, synthesis skills or critical thinking, echoing concerns in HPE literature about technology diminishing, rather than enhancing, deeper learning if not well-scaffolded.^35^ Thus, our findings suggest that AI in HPE must be intentionally integrated, with educators clarifying the nature of human-AI interaction, purpose of tasks and the limits of automation. Fears of deteriorating professional image resulting from the adoption of AI, while previously discussed in computer science and coding-heavy disciplines,^36^ surfaced spontaneously only once amongst our healthcare participants.

Barriers to adoption spanned professional, technical and organisational levels. Professionally, many participants feared degradation of clinical or educational competencies.^37^ Role confusion emerged when interprofessional boundaries become blurred by AI-enabled access to knowledge. Technically and organisationally, inadequate familiarity with AI tools, inconsistent institutional communication (who leads, what tools are endorsed), and lack of personal evidence of benefit (time saved, quality improved) inhibited uptake. These themes mirror broader healthcare AI adoption research, for instance, Hassan found trust workflow compatibility and organisational readiness were major determinants of AI uptake in healthcare.^20^ In psychiatric settings where human connection is central and metrics may be less standardised; these barriers may be accentuated.

Conversely, enablers identified in this study suggest pathways forward. Training that embeds AI use into real clinical and educational tasks supports familiarity, builds confidence and enables critique of tool output (for example, in supervision using virtual patients and AI prompts). Clear governance frameworks (data security, consent, intellectual property in content generation) can provide guidance for AI use and reduced uncertainty. Future consideration will have to be given to ensure licensing is specifically given for practitioners using AI-augmented interventions.  Peer networks and communities of practice emerged as informal but meaningful channels for sharing use-cases, overcoming resistance and co-creating norms of responsible AI use, which may be more trusted than top-down directives. These findings align with implementation research emphasising training, guidance framework and community of practice enablers for adoption of digital health tools in healthcare including psychiatric care.^38^^, ^^39^

From a practical standpoint, our findings point to potentially actionable strategies for institutions deploying AI in psychiatric care and educational settings which are generalisable to other healthcare contexts. First, plans for AI adoption and governance in clinical care should delineate clinical and operational boundaries as explicitly as possible, clarifying which tasks remain human-centric (consumer contact in clinical history taking, reviews, risk assessment, treatment decisions), which can be augmented by AI (summarisation, documentation, transcription) and how workflows change within interprofessional teams (prioritisation of time spent with consumers, with delegation of certain documentation and administrative tasks to AI). Second, training in digital literacy should include lessons about when, why and how to critically interpret AI output, emphasising the importance of evidence-based medicine, validation, reflection, synthesis and critical reasoning. Third, institutions should promote peer-led communities of practice where early adopters share prompts, workflows and lessons. This can help to bridge the divide between institutional policy and everyday use. Fourth, fostering a culture of responsible exploration and experimentation can generate “personal evidence” which many participants requested before broader adoption. This can be accomplished with small pilots that measure operational and personal outcomes (documentation time saved, learner engagement metrics, interdisciplinary workflow improvements). Finally, design of AI deployment should involve the relevant stakeholders (including data science officers, interprofessional teams many of whom are also educators) so that AI training supports actual clinical workflows and vice-versa with the aim to optimise care and education within psychiatric care settings.

### Strengths and Limitations

This study has several limitations that should be acknowledged. First, the sample consisted of 24 participants from a single tertiary psychiatry care setting. While interprofessional diversity was sought, the sample composition and setting mean findings may not reflect the full range of perspectives in other non-tertiary psychiatric care environments. Second, participants were purposefully recruited based on their involvement in both clinical care and HPE with interest in AI. This may under-represent healthcare staff who are less involved in education or less interested in AI, thus potentially biasing the findings toward more engaged views. Third, although we followed reflexivity protocols and team‐based coding,28 which helped to mitigate individual biases, the interviewer’s institutional role supporting AI and innovation may have introduced social desirability bias into the way participants presented their interest in AI. Finally, psychiatry, unlike other medical disciplines, where AI has established itself as an aid to several processes, relies heavily on language, its quality and its content, making it at once a prime discipline for the advances of LLMs but also vulnerable to their errors. This may mean that the perspectives of our participants, by virtue of their involvement in psychiatric care, may reflect views that are not transferable to other healthcare professionals.

## Conclusions

This study provides interprofessional perspectives on the integration of artificial intelligence within psychiatric care and related HPE. Across physicians, nurses, and allied health professionals, AI was viewed primarily as an adjunct to human expertise such as enhancing efficiency, documentation,^13^ and learning flexibility.^22^ Barriers included concerns about skill erosion, role confusion and limited familiarity, while enablers centred on integrated training, transparent institutional guidance and peer led community of practice. These findings highlight that effective AI adoption in psychiatric contexts requires balancing AI adoption with preservation of professional identity and patient connection. Effective AI integration in psychiatric care and education demands explicit delineation of human and AI-supported tasks, embedded digital literacy, and establishment of appropriate governance frameworks. Active engagement of relevant stakeholders and peer-led communities of practice are essential to align AI deployment with real clinical workflows, enhancing patient care and educational outcomes.

### Acknowledgements

The authors would like to thank the participants for their contribution.

### Conflict of Interest

The author**s** declare that there is no conflict of interest.
